# Modelling bodyweight to avoid anthelmintic underdosing of goats in resources-limited settings

**DOI:** 10.1007/s11250-023-03479-6

**Published:** 2023-02-09

**Authors:** M. J. Evans, C. L. Kaona, A. K. Barros, J. L. Burdon Bailey, P. Chikungwa, L. M. Costa-Junior, L. Gamble, A. M. Hopker, R. F. Kelly, F. Lohr, R. Silva, N. D. Sargison

**Affiliations:** 1 Royal (Dick) School of Veterinary Studies, University of Edinburgh, Roslin, UK; 2 Lilongwe University of Agriculture and Natural Resources, Lilongwe, Malawi; 3 Worldwide Veterinary Services, Cranborne, UK; 4 Universidade Federal Do Maranhão, São Luís, Brazil; 5 Department of Animal Health and Livestock Development, Lilongwe, Malawi

**Keywords:** Goat, Bodyweight, Anthelmintic, Sustainable development

## Abstract

Improved management of livestock in resource-limited settings can provide a means towards improved human nutrition and livelihoods. However, gastrointestinal nematodes (GIN) are a significant production-limiting factor. Anthelmintics play a role in GIN management; however, few anthelmintic classes are available in many low-middle-income countries. Utilising a limited range of classes may increase selection for anthelmintic resistance; therefore, strategies to reduce other selective pressures are of heightened importance. Avoiding anthelmintic underdosing is one such strategy, but it can be challenging without access to accurate bodyweight measurement. Many previous studies have used thoracic girth as a practical proxy for bodyweight in goats; however, they have rarely considered the potential impact of natural variation on therapeutic doses. Here, the relationship between bodyweight and thoracic girth was modelled using data from 820 goats from three Malawian biomes in two seasons, with the specific aim of avoiding underestimation of bodyweight. The internally cross-validated linear regression (∛Weight ~ 0.053 + 0.040*Girth, *R*^2^ = 0.92, rounded up to the nearest 5 kg) was validated against data from an additional 352 Malawian goats (1.4% of goats allocated an underdose and 10.2% allocated a dose > 200% of bodyweight). The equation was further externally validated using an historical dataset of 150 goats from Assam, India (2.7% of goats were allocated to an underdose and 24.8% allocated to a > 200% of bodyweight). These results suggest that a more globally generalisable approach may be feasible, provided the accuracy of the estimate is considered alongside the therapeutic index of the pharmaceutical.

**Supplementary Information:**

The online version contains supplementary material available at 10.1007/s11250-023-03479-6.

## Introduction

Small ruminant production has been identified as a potentially sustainable, climate-resilient route towards improving livelihoods in many resources-limited settings (Sargison et al. 2020). Malawi is one such example, remaining one of the world’s economically poorest nations, with approximately 50% of the population living in poverty despite recent economic growth (World Bank 2020). Approximately 80% of the population of Malawi are employed in agriculture, and this sector has been identified as particularly vulnerable to climatic shocks (World Bank 2020; Ignaciuk et al. 2021). Small ruminants (principally goats) are the most numerous mammalian livestock species in Malawi (Food and Agriculture Organization 2005). Whilst there is little research quantifying the economic impact of endemic livestock diseases in lower- and middle-income countries (LMICs) (Perry and Grace 2009), haemonchosis is a well-recognised problem across sub-Saharan Africa (Emery et al. 2016); therefore, sustainable control of this gastrointestinal nematode (GIN) infection is vital, if small ruminant production is to function as a route towards poverty alleviation (Bessell et al. 2018).

Sargison et al. (2021) demonstrated that FAMACHA scoring and faecal egg counting can be an effective way to engage Malawian smallholders with the concept of animal health management; however, that study also identified a potential lack of efficacy with albendazole treatments. In order to improve animal health and production, and to maintain farmer engagement, it is essential that the efficacy of advocated treatments is maximised. At the same time, for interventions to be sustainable longer term, it is key that the development and spread of anthelmintic resistance are minimised. This is particularly important given that benzimidazoles and ivermectin are the only anthelmintics commonly available in Malawi (author observation). Utilising a limited range of anthelmintic classes may increase selection for anthelmintic resistance, as well as reducing mitigation strategies once resistance establishes. There are very few studies investigating the presence and scale of anthelmintic resistance in LMICs and efficacy may currently be high (Seyoum et al. 2017); however, genetic analysis of *Haemonchus* spp. in Pakistan revealed evidence of multiple rapid emergences of benzimidazole resistance (Ali et al. 2019) similar to the progression previously observed in intensive production systems situated in high-income countries (Kotze et al. 2020). Affordable strategies aimed at reducing other selective pressures for anthelmintic resistance may therefore be very valuable in resource-limited settings.

One such strategy is to avoid the administration of subtherapeutic doses of anthelmintics (Smith et al. 1999). However, it is unusual for Malawian farmers to have access to weigh scales; therefore, it is necessary to develop proxy measurements that can be used to estimate goats’ bodyweight accurately. The relationships between goat bodyweight and morphometrics (particularly thoracic girth at the level of the heart) have been extensively studied with 42 worldwide examples found during a literature search for this study (Supplementary Tab.S1). However, there have been no studies utilising goats in Malawi, and Chinchilla-Vargas et al. (2018) demonstrated variation in the relationship between chest girth and bodyweight between local breed goats in neighbouring east African countries. Furthermore, the majority of studies have focussed on defining the relationships as a selective index; only four studies discuss the variation around the line of best fit (Mayaka et al. 1996; Mahieu et al. 2011; Eyduran et al. 2017; Hopker et al. 2019); and only one of these discusses the impact that this variation would have on dose rates if the relationship was used to inform dose calculations (Hopker et al. 2019). Machila et al. (2008) investigated the impact of weight estimation on dose rates in Zebu cattle; however, they optimised their weigh tape to estimate ± 20% of true bodyweight, as they were considering medicines with narrow therapeutic indices. In contrast, ivermectin and benzimidazoles have much wider therapeutic indices (Abdou and Sharkawy 2004; Foreyt 1988; Mohsen et al. 2012). It may therefore be possible to develop a weigh tape targeted to avoid underdosing with these agents whilst avoiding dangerous overdoses. However, to the authors’ knowledge, no such studies have been previously conducted.

In this manuscript, we describe the relationship between bodyweight and thoracic girth using data from 1172 goats from three Malawian biomes in two seasons, with the specific aim of developing a practical tool for estimating goat weight prior to anthelmintic treatment. Further validation utilising an historical dataset of 150 goats from Assam, India, was also used to assess if the method could be used to accurately estimate goat weight in a phenotypically different goat population.

## Materials and methods

### Data collection

Data was collected from three regions of Malawi encompassing a range of agricultural environments: Mzimba, a rural upland region in the north of Malawi with distinct wet and dry seasons; Nsanje, an arid rural lowland region of southern Malawi; and Kunthembwe, a peri-urban district close to Blantyre. A list of goat farmers in each of the three regions was provided by the local veterinary authorities, and each of these farmers was visited twice during a 12-month period: once during the wet season and once during the dry season.

At each visit, the following data were gathered: region; season; sex; body condition score, assessed by palpation of the lumbar spinal muscle and fat cover (BCS: graded 1–5); conjunctival mucous membrane colour, assessed compared to a coloured reference card (FAMACHA®: graded 1–5); girth, measured around the thorax at the level of the heart (to the nearest centimetre); and bodyweight, measured by weighing the veterinarian holding the animal in their arms on a set of bathroom scales and then subtracting the weight of the veterinarian (to the nearest 0.1 kg).

A historical dataset from a previously published study of 150 goats in Assam, India (measured using similar techniques, following a door-to-door survey) was also reserved for model testing (Hopker et al. 2019).

The Malawi field data were collected using the WVS data collection mobile application (Gibson et al. 2018).

### Computer software

Data visualisation and linear regression analysis were performed in R Studio v1.2.1335 utilising R v4.0.3 and the following R packages: “caret”, “cowplot”, and “tidyverse”. Neural network and Ridge-Lasso regression analyses were conducted in Python v3.0.

### Data cleaning and subdivision

Data points were removed where transcribing errors were suspected (girths greater than 30 cm but bodyweights of 1 kg) and where they were suspected to be from very young kids (girths < 15 cm). This resulted in the removal of two data points from the Malawian dataset and one from the Assamese dataset.

The Malawian dataset was then divided randomly in a 70/30 split, with 70% of the data used for model training and validation. The remaining 30% of the Malawian data and all the Assamese data were reserved for subsequent model testing. (A conservative 70/30 split was utilised as preliminary analysis suggested that the variances between the two splits began to diverge just beyond 80/20.)

### Data modelling

Three modelling techniques were then employed in order to try to optimise model performance by balancing improved accuracy in the training dataset against increased complexity and risks of over-fitting (reduced generalisability). Firstly, a linear model was constructed using all six available predictor variables (region, season, sex, BCS, FAMACHA, and girth). This model was then simplified by stepwise removal of variables using *k*-fold internal cross-validation (*k* = 10) to estimate root mean squared error (RMSE) and *r*^2^. Secondly, a Ridge-Lasso regression analysis was performed using all six predictor variables. Finally, a neural network multilayer perceptron regression analysis was performed using all six predictor variables.

### Converting the model into a weigh tape

In order to avoid treatments that may compromise animal health and select for anthelmintic resistance, the final model was used to generate prediction intervals for confidence levels ranging in 0.1% increments from 1 to 99.9%. The upper bounds of these prediction intervals were then rounded up to the nearest 5 kg for practical purposes. For example, 5 kg corresponds to 0.5 ml of a 100 mg/ml solution of fenbendazole or albendazole administered orally at 10 mg/kg or 2.5 ml of an 800 µg/ml solution of ivermectin given orally at 400 µg/kg. The optimal value was then assessed by visually examining the proportion of goats with residual percentages (the percentage of their true bodyweight they would be “treated for”) falling into three bins: 0–100%; 100–200%, and over 200%.

### Testing model performance

Model performance in Malawian goats was tested by applying the weigh tape function to the reserved 30% portion of the original dataset and then calculating the residual percentages. Generalisability to a phenotypically different goat population was investigated by applying the same procedure to the Assamese dataset.

## Results

Across the two visits, a total of 1174 goats were examined. The age of goats examined ranged from kids less than a week old to adult goats. However, the vast majority of goat owners were unable to provide the age of their goat due to the frequent buying, selling, and trading of goats without written records. Age was therefore excluded from the modelling.

**Fig. 1:**
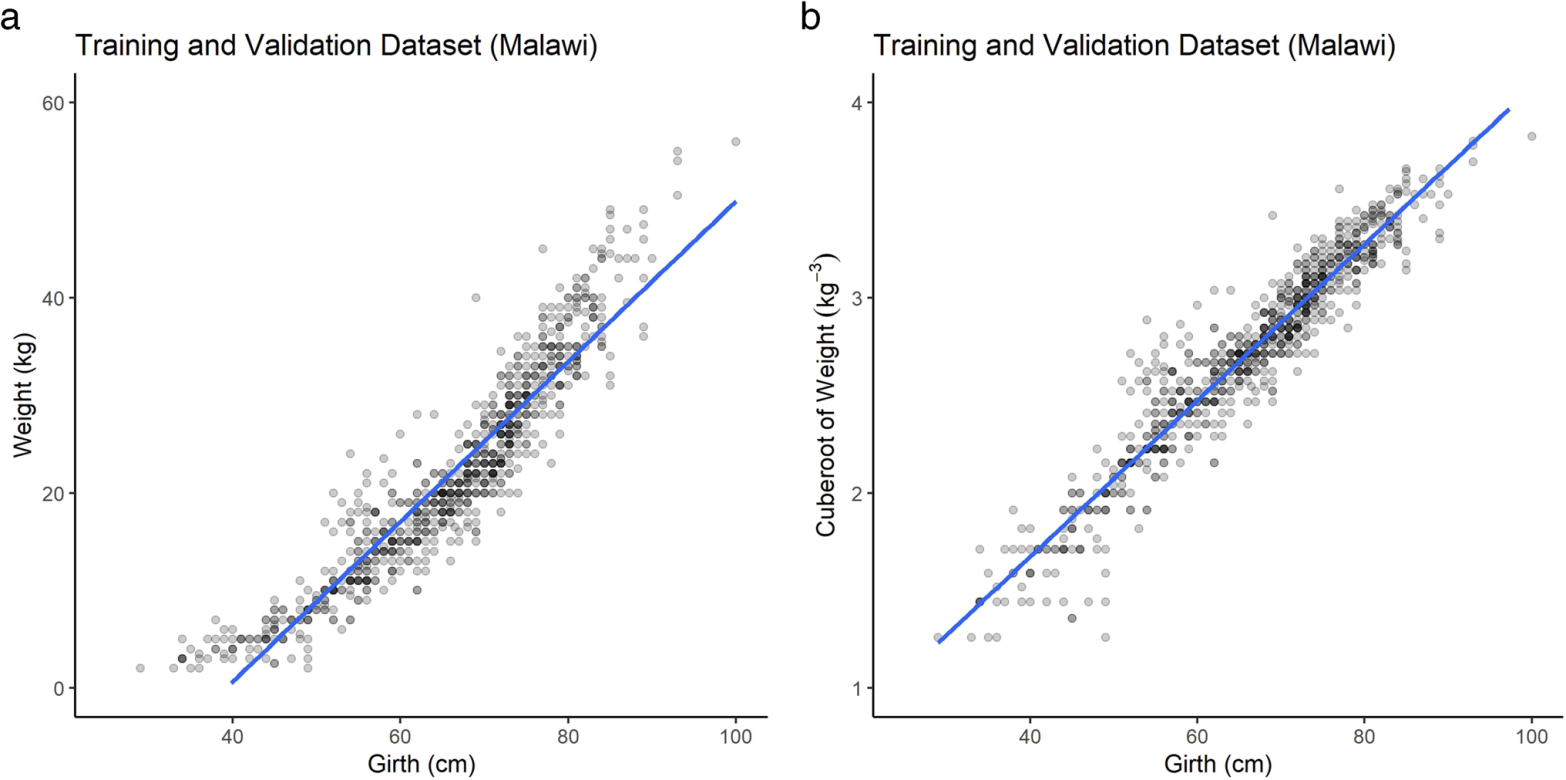
Data prior to (**a**) and subsequent to (**b**) cube root transformation. Data points are presented with 80% transparency to aid visualisation of overlying points

Visual examination of the data suggested that the relationship between girth and bodyweight was non-linear; however, an intuitive cube root transformation of bodyweight yielded a linear relationship (Fig. 1). These transformed data were therefore used for all modelling. Whilst cube-transforming girth would yield the same linear relationship, we preferred not to transform girth in order to maintain the clarity of the relationship between the model and the subsequent weigh tape.

Stepwise simplification of the linear regression yielded extremely similar values for mean *R*^2^ and mean RMSE irrespective of the number of variables included (Table 1). In order to avoid unnecessary complexity, the univariate linear model (∛Weight ~ 0.053 + 0.040*Girth, *R*^2^ = 0.92) was therefore chosen as the optimal model at this stage.

**Table 1:** Results of stepwise linear model simplification with internal cross-validation (10 folds)

Variables	Model call	Mean *R*^2^	Mean RMSE
6	∛Weight ~ Sex + Girth + Region + BCS + FAMACHA + Season	0.922	0.143
5	∛Weight ~ Sex + Girth + Region + FAMACHA + Season	0.921	0.144
4	∛Weight ~ Sex + Girth + Region + Season	0.922	0.144
3	∛Weight ~ Girth + Region + Season	0.920	0.144
2	∛Weight ~ Girth + Season	0.923	0.145
1	∛Weight ~ Girth	0.919	0.146

The Ridge-Lasso regression method yielded a model with an *R*^2^ value of 0.93, and the neural network analysis yielded a model with an *R*^2^ value of 0.91. As these values were so similar to the linear regression results, these more complex methods were discarded, and the univariate linear regression was carried forward as the final model.

**Fig. 2:**
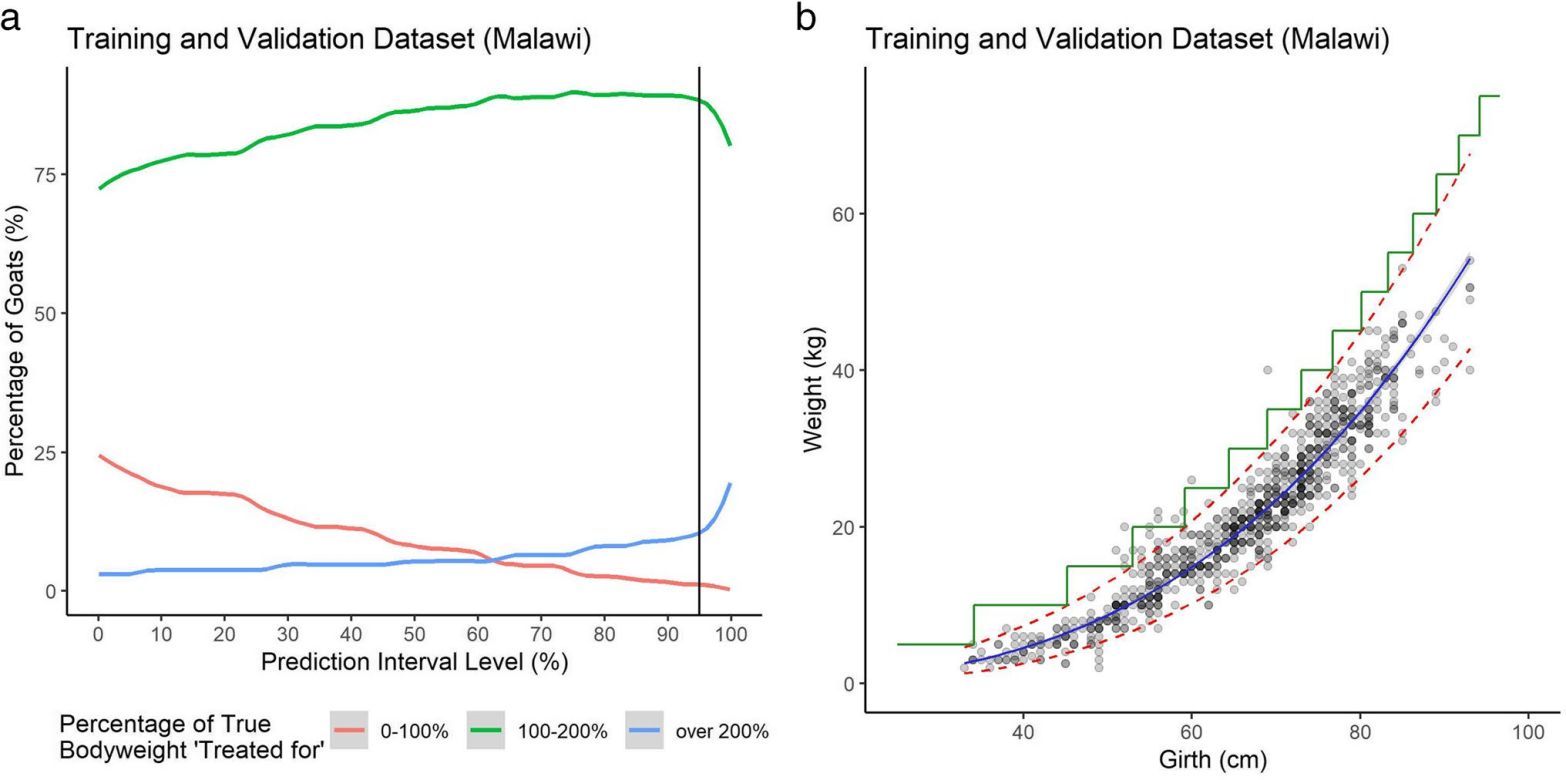
**a** The percentage of goats in the training and validation dataset that would be “treated for” either 0–100%, 100–200%, or over 200% of their true bodyweight, plotted against the confidence level from which the upper bound of the prediction interval was obtained (after rounding up to the nearest 5 kg). The selected 95% prediction interval values are shown by the vertical line. **b** Plot of weight (back-transformed) against girth. Data points are presented with 80% transparency to aid visualisation of overlying points. The regression line is shown in the centre of the data points (blue in online colour version), with its 95% confidence interval a narrow grey band. The upper and lower bounds of the 95% prediction interval are shown by the dashed lines (red in online colour version). The weigh tape function is shown by the solid stepped line (green in online colour version)

Visual assessment of the impact of altering the prediction interval confidence level (with rounding) on the percentage residuals suggested that at a confidence level of approximately 95%, there was an inflection point in the proportion of goats being “treated for” over 200% of their bodyweight (Fig. 2A). Therefore, the upper bound of the 95% prediction interval (rounded up to the nearest 5 kg) was utilised for the weigh tape to avoid underdosing (Fig. 2B and Table 2).

**Table 2:** Weigh tape conversion table

Girth (cm)	Dose for (kg)
15–36	5
37–45	10
46–52	15
53–58	20
59–63	25
64–68	30
69–72	35
73–76	40
77–80	45
81–84	50
85–87	55
88–91	60
92–94	65
95–97	70
98–100	75

**Fig. 3:**
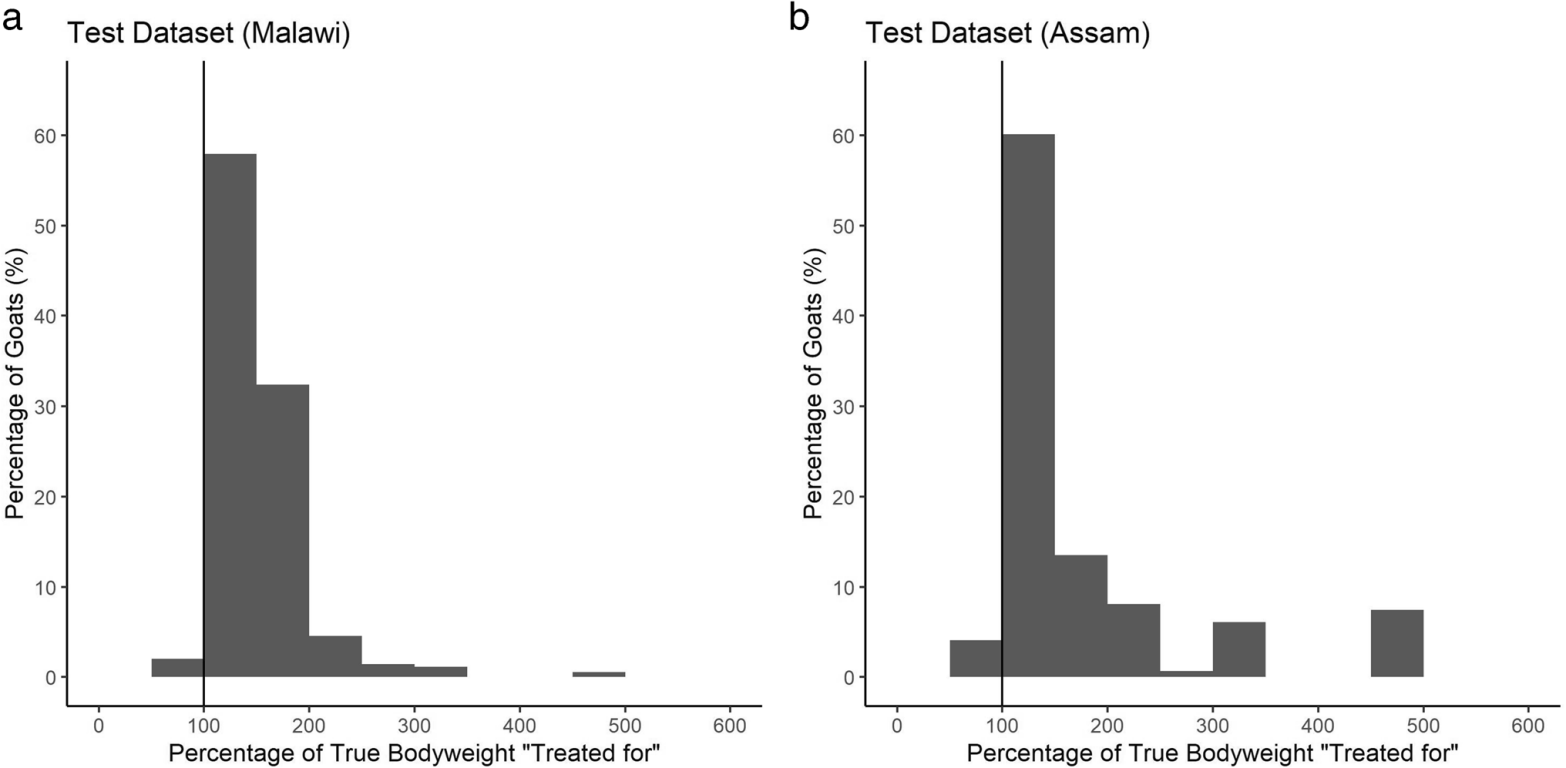
Percentage residuals (what the goats would have been “treated for” as a percentage of their true bodyweight) for the Malawian test dataset (**a**) and the Assamese dataset (**b**)

When the weigh tape function was applied to the Malawian test dataset, performance was excellent, with only 1.4% of goats being allocated to an underdose (less than 100% of bodyweight) and 10.2% of goats being allocated to a dose of greater than 200% of bodyweight (Fig. 3A). When the function was extended to the Assamese test dataset, performance was reasonable, although only 2.7% of goats were allocated to an underdose and 24.8% of goats being allocated to a dose of greater than 200% of bodyweight (Fig. 3B). In both test datasets, the maximum predicted overdose was 500% of bodyweight (Fig. 3).

## Discussion

As has been previously described in other regions (see Tab. S1), thoracic girth was shown to be an accurate proxy measure for bodyweight in Malawian goats. However, given that the residuals of the regression model were normally distributed, if the regression line alone was used to inform treatment, approximately 50% of goats would be treated for less than 100% of their bodyweight. This variation around the regression has rarely been considered in previous studies, which have commonly focussed on predictors of bodyweight that may be used for selective breeding programmes. However, the variation reported here is wide, justifying the need to consider it when making therapeutic considerations, particularly given underdosing is a well-recognised risk for the development of anthelmintic resistance (Smith et al. 1999).

However, optimising the weigh tape to avoid underdosing inevitably led to an increased proportion and magnitude of hypothetical overdoses relative to bodyweight. Although there are limited studies in goats, the therapeutic indices for benzimidazoles and ivermectin are likely wide, as a 7.5-fold overdose of albendazole showed no adverse effects in goat kids (Foreyt 1988) and the ivermectin toxicoses in goats reported by Abdou and Sharkawy (2004) and by Mohsen et al. (2012) were for 10–20-fold overdoses. The relative overdoses predicted in the overwhelming majority of the Malawian test dataset goats in this study would arguably not have been of clinical concern. However, this is obviously not true for all therapeutics, and extreme caution should be applied before extrapolating these results to other actives with narrower therapeutic indices (e.g. levamisole in goats). It would be ideal to optimise the weight estimation according to the therapeutic index for each drug; however, therapeutic indices are unfortunately not available for many goat medicines.

It is not possible to judge whether the small number of goats with outlying bodyweight to girth relationships were true anatomic extremes or the result of data errors. When this weigh tape is applied in the field, it will therefore be necessary to introduce a degree of sense checking to avoid potentially dangerous overdoses (perhaps estimating the weight visually prior to using the weigh tape and applying clinical judgement if the weigh tape recommendation would result in a dangerous overdose relative to the prior estimate). Smallholder and veterinary/paraveterinary education in this regard may therefore be a valuable component to future field validation (Machila et al. 2007; Bessell et al. 2017).

It is interesting that no significant differences in the relationship between girth and bodyweight were observed between the regions of Malawi in this study, given that differences between neighbouring east African countries have been observed (Chinchilla-Vargas et al. 2018) and there have been efforts to improve the native goats in Mzimba with Boer genetics. This suggests that the weigh tape is applicable across the Malawian biomes studied here. It is not surprising that model performance was poorer when applied to the Assamese dataset, given that the goats in Assam are very different in body shape from those in Malawi (the Assamese goats are shorter and more rotund). The results of applying the function to the Assamese data suggest that utilising this weigh tape there would be relatively unlikely to lead to underdosing; however, there would be a greater degree of overdosing. It would therefore be valuable to run field validation trials in multiple locations with varying breeds of goats, before utilising this weight tape outside of Malawi (and cautiously in Assam). Given the many prior studies investigating links between goat morphometry and bodyweight, there is an opportunity for meta-analysis to expand the generalisability of models such as that described here; however, it is limited by data accessibility.

It was not possible to model to the effect of age on the girth to bodyweight relationship in this study, as accurate age data was not available. Age has been suggested as a helpful additional metric for adjusting weight estimation in goats (Mahieu et al. 2011; Hopker et al. 2019), and it is vital context if weight is to be used as a proxy for performance when informing breeding or animal health decision making. The introduction of transferrable records that allow the age of goats to be passed on following changes in ownership would therefore also be valuable.

If further data suggests more complex models would be valuable (e.g. including, region, breed, BCS), the machine learning techniques trialled in this study may prove more valuable. That would increase the computational complexity of the models; however, that could be overcome with a relatively simple mobile application (if smartphone access is available). Such an application could also feed data back into the opensource database (as has been achieved with the citizen science “INaturalist” application (Van Horn et al. 2018)), potentially generating a valuable repository of clinical information in settings with limited literature bases.

Of course, it is vital that clinical interventions are targeted at areas most important to the livestock farmers in the regions of interest and that are likely to have the greatest positive impacts. This study was conceived partly following high levels of anaemia and the possible lack of efficacy of albendazole in Malawi; however, there are many potential causes of anaemia in small ruminants in sub-Saharan Africa (Sargison et al. 2021). There is therefore also a need to further describe the animal health challenges faced by smallholders in LMICs and quantify how interventions impact production and livelihoods in the field.

In conclusion, there is a clear need for proxy measures to enable the treatment of helminth infections of livestock in resource-limited settings that avoid ineffectual and resistance-selecting underdoses whilst considering the risks of overdosing. Thoracic girth provides a suitable such proxy in goats in Malawi, with the results of this study generating a weigh tape that would be suitable for informing benzimidazole or ivermectin treatments. However, high levels of relative overdoses on a bodyweight basis indicate the need for caution if using bodyweight proxies for actives with narrower therapeutic indices. The application of this weigh tape to a historical dataset from Assam, India, suggests there is potential for a more globally generalisable technique, although there is a need to pool research efforts from as many geographical settings as possible.

## Supplementary Information

Below is the link to the electronic supplementary material.

Supplementary file1 (DOCX 35 KB)

Supplementary file2 (CSV 45 KB)

## Data Availability

The data collected in this study are provided in supplementary file S1—the authors are committed to open access data and would encourage its reuse, after contact to discuss collaborative opportunities. The data provided from the Assamese goats may be requested by contacting the authors of that study (Hopker et al. 2019).

## Declarations

### Disclaimer

For the purpose of open access, the author has applied a CC-BY public copyright licence to any Author Accepted Manuscript version arising from this submission.
