# Supplementary material for: Modelling bodyweight to avoid anthelmintic underdosing of goats in resources-limited settings
Source: Trop Anim Health Prod. 2023 Feb 9;55(2):72. doi: 10.1007/s11250-023-03479-6 (PMC9911490; doi:10.1007/s11250-023-03479-6)
Supplement: Supplementary file 1 — Supplementary file1 (DOCX 35 KB) [file 11250_2023_3479_MOESM1_ESM.docx]

# Supplementary Table S1

Literature search

Database: CAB Abstracts

Search terms: Goat AND Girth AND Weight; Goat AND Girth AND Bodyweight

Selected for relevance from abstract

| **Author(s)** | **Title** | **Reference** |
| --- | --- | --- |
| Abdel-Mageed, I. and Ghanem, N. | Predicting body weight and longissimus muscle area using body measurements in subtropical goat kids. | Egyptian Journal of Sheep and Goat Sciences; 2013. 8(1):95-100. 22 ref. |
| Adeyinka, I. A. and Mohammed, I. D. | Accuracy of body weight prediction in Nigerian Red Sokoto goats raised in North Eastern Nigeria using linear body measurement. | Pakistan Journal of Biological Sciences; 2006. 9(15):2828-2830. 17 ref. |
| Akingbade, A. A. et al. | Relationships between heart girth and body weight measurements of South African indigenous Nguni goats. | Indian Journal of Animal Sciences; 2003. 73(10):1141-1143. 16 ref. |
| Ashraf Waiz, H and Gautam, L. | Determination of best fitted regression model for assessment of live body weight from morphological traits in Sirohi goat in Udaipur. | Indian Journal of Animal Sciences; 2020. 90(5):804-808. 15 ref. |
| Badi, A. M. I. et al. | Estimation of live body weight in Eritrean goat from heart girth and height at withers. | Indian Journal of Animal Sciences; 2002. 72(10):893-895. 11 ref. |
| Bello, A. A. and Adama, T. Z. | Studies on body weight and linear body measurements of castrates and non-castrate savannah brown goats. | Asian Journal of Animal Sciences; 2012. 6(3):140-146. 30 ref. |
| Birteeb, P. T. and Lomo, R. | Phenotypic characterization and weight estimation from linear body traits of West African Dwarf goats reared in the transitional zone of Ghana. | Livestock Research for Rural Development; 2015. 27(9):Article 175. 26 ref. |
| Cam, M. A. et al. | Possibilities of using morphometrics characteristics as a tool for body weight prediction in Turkish Hair Goats (Kilkeci). | Asian Journal of Animal and Veterinary Advances; 2010. 5(1):52-59. 29 ref. |
| Chinchilla-Vargas et al. | Predicting live weight of rural African goats using body measurements | Livestock Research for Rural Development; 2018. 30(7):123 |
| Chitra, R. et al. | Prediction of body weight using appropriate regression model in adult female Malabari goat. | Veterinary World; 2012. 5(7):409-411. 12 ref. |
| Costa, E. de O. et al | Morphometric measurements associated with mathematical prediction of body weight as a management tool in goat herds. | Archivos de Zootecnia; 2020. 69(268):454-460. 26 ref. |
| Dorantes-Coronado, E. J. et al. | Zoometric measures and their utilization in prediction of live weight of local goats in southern Mexico. | SpringerPlus; 2015. 4(695):(12 November 2015). 24 ref. |
| Eyduran, E. et al. | Comparison of the predictive capabilities of several data mining algorithms and multiple linear regression in the prediction of body weight by means of body measurements in the indigenous Beetal goat of Pakistan. | Pakistan Journal of Zoology; 2017. 49(1):273-282. 30 ref. |
| Eyduran, E. et al. | Prediction of live weight from morphological characteristics of commercial goat in Pakistan using factor and principal component scores in multiple linear regression. | JAPS, Journal of Animal and Plant Sciences; 2013. 23(6):1532-1540. 16 ref. |
| Feyissa, A. A. et al. | Application of body measurements of blackhead Somali sheep as parameters for estimation of live weight. | Iranian Journal of Applied Animal Science; 2018. 8(4):647-652. 32 ref. |
| Gul, S. et al. | Some prediction equations of live weight from different body measurements in Shami (Damascus) goats. | Journal of Animal and Veterinary Advances; 2005. 4(5):532-534. 6 ref. |
| Hayashi, Y. et al. | Feeding practice and bodyweight estimation of goat in Chitwan district of Nepal. | Nepalese Journal of Agricultural Sciences; 2015. 13:220-225. 13 ref. |
| Hopker, A. et al. | Weight estimation in native crossbred Assamese goats. | Livestock Research for Rural Development; 2019. 31(10):Article 162. 21 ref. |
| Khargharia, G. et al. | Relationship of body weight with linear body measurements of Assam Hill goat using path analysis. | Indian Journal of Animal Production and Management; 2015. 31(1/2):unpaginated. 13 ref. |
| Khorshidi-Jalali, M. et al. | Comparison of artificial neural network and regression models for prediction of body weight in Raini Cashmere goat. | Iranian Journal of Applied Animal Science; 2019. 9(3):453-461. 41 ref. |
| Mahieu, M. et al. | Predicting the body mass of goats from body measurements. | Livestock Research for Rural Development; 2011. 23(9):192. 28 ref. |
| Matsebula, M. et al. | Prediction of live weight from linear body measurements of indigenous goats of Swaziland. | Livestock Research for Rural Development; 2013. 25(8):Article 140. 18 ref. |
| Mayaka, T. B. et al. | Estimation of live body weight in West African Dwarf goats from the heart girth measurement. | Tropical Animal Health and Production; 1996. 28(1):126-128. 9 ref. |
| Moaeen-ud-Din, M. et al. | Evaluation of different formulas for weight estimation in Beetal, Teddi and Crossbred (Beetal x Teddi) goats. | JAPS, Journal of Animal and Plant Sciences; 2006. 16(3/4):70-74. 27 ref. |
| Mohammed, I. D. and Amin, J. D. | Estimating body weight from morphometric measurements of Sahel (Borno White) goats. | Small Ruminant Research; 1997. 24(1):1-5. 17 ref. |
| Moses, O. D. | Application of factor analysis scores in a multiple linear regression model for the prediction of liveweight in immature West African dwarf goat. | Philippine Journal of Veterinary and Animal Sciences; 2010. 36(2):167-174. |
| Mule, M. R. et al. | Relationship of body weight with linear body measurement in Osmanabadi goats. | Indian Journal of Animal Research; 2014. 48(2):155-158. 18 ref. |
| Netsanet Zergaw et al. | Using morphometric traits for live body weight estimation and multivariate analysis in Central Highland and Woyto-Guji goat breeds, Ethiopia. | African Journal of Agricultural Research; 2017. 12(15):1326-1331. 20 ref. |
| Norris, D. et al. | Path coefficient and path analysis of body weight and biometric traits in indigenous goats. | Indian Journal of Animal Research; 2015. 49(5):573-578. |
| Pander, B. L. et al. | Growth performance and prediction of body weight from body measurements in Beetal and Black Bengal kids and their crosses maintained under feed lot conditions. | Indian Journal of Animal Production and Management; 1989. 5(4):162-166. 5 ref. |
| Pares, P. M. et al. | Live weight estimation of gwembe goat (Capra hircus) from measurement of thoracic girth. | Journal of Veterinary Anatomy; 2012. 5(2):9-14. 9 ref. |
| Perez, Z. O. et al. | Body weight estimation using body measurements in goats (Capra hircus) under field condition. | Philippine Journal of Veterinary and Animal Sciences; 2016. 42(1):1-7. |
| Rideout, C. B. and Worthen, G. L. | Use of girth measurement for estimating weight of mountain goats. | Journal of Wildlife Management; 1975. 39(4):705-708. 22 ref. |
| Rotimi, E. A. et al. | Relationship between bodyweight and morphological traits in Sahelian goats of Nigeria using path analysis. | Mustafa Kemal Universitesi Tarim Bilimleri Dergisi; 2020. 25(3):455-460. 27 ref. |
| Semakula, J. et al. | Variability in body morphometric measurements and their application in predicting live body weight of Mubende and small East African goat breeds in Uganda. | Middle East Journal of Scientific Research; 2010. 5(2):98-105. 28 ref. |
| Senda, T. S. et al. | Live weight estimation in the indigenous matebele goats using the heart girth circumference. | Scientific Journal of Animal Science; 2014. 3(12):301-304. 11 ref. |
| Sowande, O. S. et al. | Age- and sex-dependent regression models for predicting the live weight of West African Dwarf goat from body measurements. | Tropical Animal Health and Production; 2010. 42(5):969-975. 25 ref. |
| TekIn, M. E. et al. | Determination of relationship of body weight and some body measurements by nonlinear models in hair goats in Karaman region. | Eurasian Journal of Veterinary Sciences; 2019. 35(2):99-103. 8 ref. |
| Tsegaye, D. et al. | Linear body measurements as predictor of body weight in Hararghe highland goats under farmers environment: Ethiopia. | Global Veterinaria; 2013. 11(5):649-656. 29 ref. |
| Tyagi, K. K. et al. | Determination of age and sex dependent best-fitted regression model for predicting body weight in Surti kids. | Indian Journal of Small Ruminants; 2013. 19(1):79-82. 7 ref. |
| Waheed, H. M. et al. | Prediction of monthly body weight from body measurements in Beetal goats reared under field and farm conditions. | JAPS, Journal of Animal and Plant Sciences; 2020. 30(1):25-31. 24 ref. |
| Yakubu, A. | Fixing collinearity instability in the estimation of body weight from morpho-biometrical traits of West African dwarf goats. | Trakia Journal of Sciences; 2009. 7(2):61-66. 23 ref. |
